# Disseminated Metastatic Renal Cell Carcinoma Manifesting With Recurrent Epistaxis: A Case Report and Comprehensive Literature Review

**DOI:** 10.1155/crot/7461155

**Published:** 2025-06-12

**Authors:** Andrew J. Rothka, David Goldrich, Sanica Bhele, Johnathan D. McGinn

**Affiliations:** ^1^Penn State College of Medicine, Hershey, Pennsylvania, USA; ^2^Department of Otolaryngology-Head and Neck Surgery, Penn State Health Milton S. Hershey Medical Center, Hershey, Pennsylvania, USA; ^3^Department of Pathology, Penn State Health Milton S. Hershey Medical Center, Hershey, Pennsylvania, USA

## Abstract

Renal cell carcinoma is an aggressive malignancy with up to 30% of patients experiencing metastases. The authors report a case of a patient status post right radical nephrectomy with 6 years of clear surveillance scans seeking evaluation of recurrent epistaxis. A friable, hypervascular mass was discovered on outpatient nasal endoscopy. The mass was surgically removed, and pathology results were consistent with metastatic renal cell carcinoma. Further workup following the operation led to the discovery of disseminated metastases of the malignancy to the scrotum, skin of the back, gluteal musculature, and frontal bones. This unique case of disseminated metastases after many years of negative routine screening demonstrates the importance of interdisciplinary care and routine screenings when managing unforgiving malignancies such as renal cell carcinoma and their insidious manners of metastasis.

## 1. Introduction

Renal cell carcinoma (RCC) is an aggressive malignancy with metastasis reported to occur in upwards of 30% of patients [1]. Metastasis has been shown to appear even more than 10 years following nephrectomy, which emphasizes the importance of routine surveillance [2]. The authors present a case of a patient with a history of RCC with negative annual screens who developed recurrent epistaxis secondary to a nasal mass, which led to further workup and discovery of disseminated metastases.

## 2. Case Presentation

A 56-year-old male presented to the Otolaryngology outpatient clinic for evaluation of recurrent epistaxis and rhinorrhea. The patient had a past medical history significant for RCC status post open right radical nephrectomy 6 years prior, hypertrophic cardiomyopathy, mitral and tricuspid regurgitation, and sarcoidosis. His last annual screen with his urologist was performed 4 months before presenting with epistaxis. CT imaging at that time displayed no evidence of metastasis or recurrence. The epistaxis occurred from the right nostril approximately 3 times per week and was easily controlled with conservative measures. Occasionally, blowing his nose led to an episode of epistaxis. The patient denied prior a history of epistaxis before this presentation. He noted increased discolored mucus production from the right. He denied unintentional weight loss, fevers, chills, and night sweats. The patient was an active cigarette smoker with an estimated 20 pack-year history.

**Figure 1:**
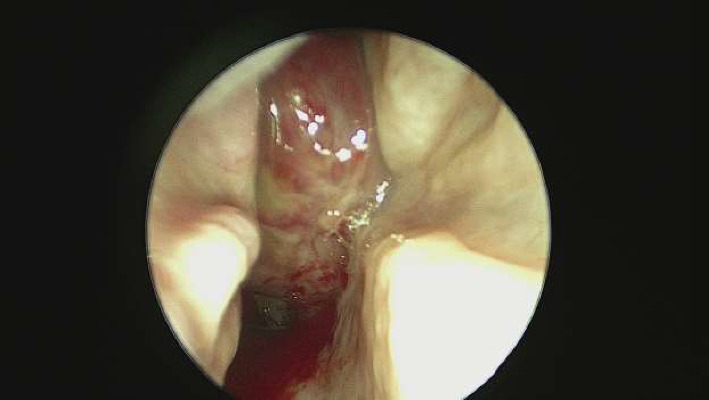
Outpatient endoscopy of right nasal passage.

Outpatient endoscopy of the right nasal passage (Figure 1) revealed thick purulent secretions starting at the heads of the middle and superior turbinate with a red mass in the posterior nasal passage, which appeared to emanate from the lateral nasal wall beneath the middle turbinate attachment. The mass occupied a majority of the nasal cavity posteriorly and extended into the nasopharynx. It appeared hypervascular with significant blood vessels along its surface. The left nasal passage and nasopharynx revealed no abnormalities.

**Figure 2:**
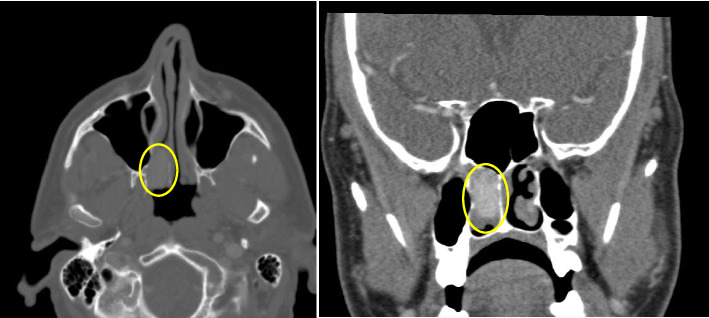
CT sinus of the sinonasal mass on presentation. The lesion is outlined in each image with a yellow circle.

The differential diagnosis based on endoscopic findings included primary sinonasal neoplasms, such as hemangiopericytoma, inverted papilloma, and mucosal melanoma, as well as RCC metastasis. A computed tomography (CT) sinus was ordered with and without contrast to further determine the extent of the nasal mass (Figure 2). Results of the CT showed a homogeneously hyperdense enhancing, well-defined mass in the right nasal cavity measuring approximately 1 × 2 × 2 cm, without bony erosion, favoring a less aggressive neoplasm. A sinonasal malignancy was considered less likely at that time.

During his outpatient workup of this nasal mass, the patient presented to the Emergency Department (ED) with an erosive lesion on his scrotum. He underwent local excision of the scrotal lesion with Urology in the ED, and surgical pathology displayed discrete nests of tumor cells with optically clear cytoplasm with a delicate branching vascular pattern, consistent with classic clear-cell RCC.

**Figure 3:**
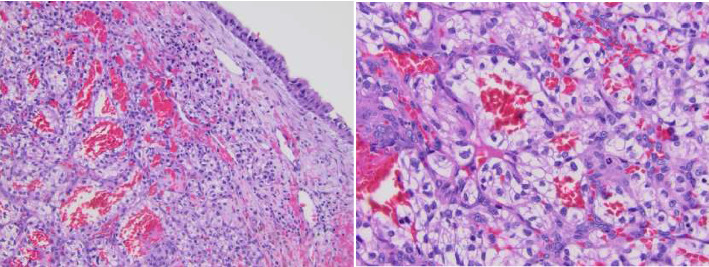
Surgical pathology slides of the nasal mass.

The patient was scheduled for endoscopic surgery with right endoscopic maxillary antrostomy, possible ligation of the sphenopalatine artery, and resection of the nasal mass for diagnostic purposes as well as to control his recurrent epistaxis events. The patient underwent the scheduled procedure without complications. Surgical pathology was also consistent with metastatic clear-cell RCC (Figure 3).

**Figure 4:**
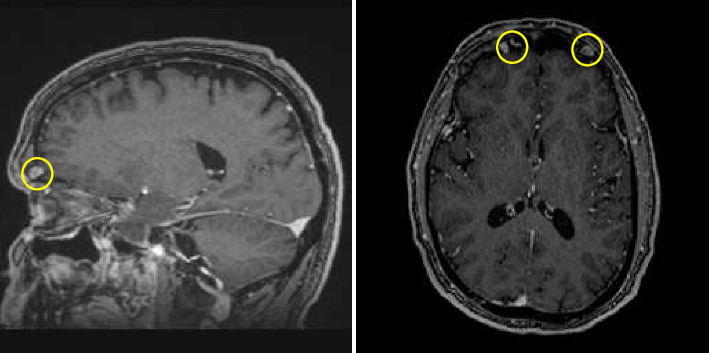
MRI of the brain in sagittal and axial views. The lesions are outlined with yellow circles.

Further workup included magnetic resonance imaging (MRI) brain with and without contrast (Figure 4) and full-body positron emission tomography (PET) CT, with findings including enhancing lytic lesions in the right and left frontal bone abutting the frontal lobe, extensive hilar and mediastinal lymphadenopathy concerning for sarcoidosis versus metastasis, increased uptake in the right gluteal musculature, and diffuse bladder wall thickening. Multiple cutaneous lesions were discovered on the patient's back, which were confirmed to be metastases after biopsies were performed. The patient was referred to the Hematology/Oncology service for further management of his metastatic disease.

The patient is currently receiving lenvatinib therapy for his metastatic disease. He is scheduled to undergo immune checkpoint inhibitor therapy with pembrolizumab versus everolimus. Given his history of hypertrophic cardiomyopathy with atrial flutter, a cardiac MRI is being ordered prior to starting additional therapies.

## 3. Discussion

Sinonasal malignancies typically present with nonspecific symptoms such as nasal obstruction, facial pain, headache, proptosis, diplopia, impaired vision, epistaxis, and cranial nerve palsies [3]. These malignancies account for less than 5% of all head and neck neoplasms, although the exact incidence of metastasis to the sinonasal tract is unknown [4, 5]. After a comprehensive review of the literature, a well-defined incidence of metastatic malignancies to the sinonasal cavities has not been reported, although certain cancers have been reported to do such more often. Imaging modalities such as CT sinus and MRI can be utilized to further determine the extent of the mass once discovered on endoscopy [6]. Full-body images such as PET CT, as ordered for this patient, can help determine extent of further metastases.

Prognosis for patients presenting with metastatic lesions within the nose, sinonasal cavity, and nasopharynx is typically poor as it indicates disseminated disease [7]. Though a rare phenomenon in general, metastatic malignancies of the sinonasal passages are most commonly kidney, breast, lung, thyroid, liver, colon, and prostate [7, 8]. Patients with primary or metastatic sinonasal malignancies tend to display symptoms such as obstruction and epistaxis once the disease has become advanced [9]. The pathway of metastasis for RCC is poorly understood, especially to the sinonasal passages. One paper hypothesizes the spread from renal vasculature through the vertebral venous plexus as it communicates with both the great venous plexus of the head and the plexus of the paranasal sinuses [8]. More typical sites for RCC metastasis include the lungs, soft tissues, bone, liver, cutaneous sites, and central nervous system [10]. However, although rare, RCC has been cited in the literature as one of the most common malignancies to spread to the head and neck [8, 11].

While metastasis of RCC to the sinonasal passages has been more extensively reported, a comprehensive literature review identified only 9 cases of patients with metastatic RCC primarily manifesting with epistaxis. These patients are presented in Table 1 [12–20].

Patients ranged from 42 to 73 years old with a mean age of 56 years. Time from nephrectomy to discovery of metastasis ranged from 2 to 17 years postoperation with a mean time of 6.9 years. To our knowledge, the current report is the only patient with a negative screening within months of the metastasis manifesting. One patient had a history of a prior metastasis but declined further intervention at that time. The remaining studies did not comment on the patients' screenings.

The patient described in this report has widespread metastases to unique locations that presented with insidious onset. One such site is the gluteal musculature, which was previously described only once in the literature [21]. Though infrequent, renal malignancies are the most common urologic malignancy to metastasize to the skin [22]. However, there is only one published case cutaneous scrotal metastasis of RCC [23]. These widespread findings necessitated the involvement of additional specialties outside the scope of practice for the otolaryngologist and urologist, emphasizing the need for interdisciplinary care for this patient.

Interdisciplinary collaboration is a crucial aspect of patient-centered care when monitoring aggressive malignancies such as RCC. Multiple, prompt referrals made on behalf of patients allow for early recognition of metastatic lesions. One such method for effective interdisciplinary care is a multidisciplinary tumor board. Previous studies illustrate the utility of tumor boards to effectively manage malignancies, improve clinical decision-making, and increase patient satisfaction [24, 25]. The extent of this patients' disease was discovered due to the discussions had and decisions made at a multidisciplinary tumor board. Perspectives from a wider breadth of healthcare experts can review the patient holistically and tailor the plan-of-care to the individual patients' goals of care.

## 4. Conclusion

Metastasis of RCC to the sinonasal cavity is rare and typically indicative of advanced disease. This unique case of disseminated metastases in a patient with years of negative screening emphasizes the value of routine screens and the requirement of prompt interdisciplinary care and effective communication in treating patients with past medical histories significant for aggressive malignancies such as RCC.

## Figures and Tables

**Table 1:** Patients with multiple metastases of renal cell carcinoma presenting with epistaxis.

Source	Age	Sex	Presenting symptoms	Site of sinonasal metastasis	Additional sites of metastases	Time to metastases from nephrectomy (years)
Current	56	Male	Recurrent epistaxis and rhinorrhea	Right nasal cavity	Scrotum, gluteal musculature, bilateral frontal bones, cutaneous lesions, hilar and mediastinal lymph nodes, bladder wall	6
Nayak et al. [12]	56	M	Unilateral epistaxis, facial pain, headache	Sphenoid sinus with expansion into sella, cavernous sinus, internal carotid artery, ethmoid sinus, orbit, nasal cavity, maxillary sinus	Right kidney, right adrenal gland, liver, lungs	7
Hess et al. [13]	73	M	Unilateral epistaxis, unilateral nasal obstruction	Nasal cavity, ethmoid sinus, maxillary sinus	Temporal lobe, adrenal gland	3
Sikdar et al. [14]	60	F	Unilateral epistaxis	Nasal cavity	Lung	3
Nason and Carrau [15]	56	M	Unilateral epistaxis, nasal obstruction	Nasal cavity, nasopharynx, maxillary sinus, ethmoid sinus	Thyroid	2
Ziari et al. [16]	47	M	Epistaxis, nasal obstruction, back pain	Nasal cavity, ethmoid sinus	Thoracic spine	17
Kumar et al. [17]	42	M	Epistaxis, nasal obstruction, purulent nasal discharge, melena	Nasal cavity, ethmoid sinus, frontal sinus	Lungs	10
Doğan et al. [18]	45	F	Epistaxis, nasal obstruction	Nasal cavity, ethmoid sinus, nasal septum	Lungs, adrenal gland	6
Parida [19]	58	F	Epistaxis	Ethmoid sinus, sphenoid sinus, intracranial extension, intraorbital extension	Kidney	10
Hefer et al. [20]	67	F	Epistaxis, nasal obstruction	Nasal cavity	Adrenal gland^∗^	5

^∗^Adrenal metastasis was discovered 2 years prior, but the patient declined further therapy.

## Data Availability

Data sharing is not applicable to this article as no datasets were generated or analyzed during the current study.

## References

[1] Chandrasekar T., Klaassen Z., Goldberg H., Kulkarni G. S., Hamilton R. J., Fleshner N. E. (2017). Metastatic Renal Cell Carcinoma: Patterns and Predictors of Metastases-A Contemporary Population-Based Series. *Urologic Oncology: Seminars and Original Investigations*.

[2] Abara E., Chivulescu I., Clerk N., Cano P., Goth A. (2013). Recurrent Renal Cell Cancer: 10 Years or More After Nephrectomy. *Canadian Urological Association Journal*.

[3] Abdelmeguid A. S., Bell D., Hanna E. Y. (2019). Sinonasal Undifferentiated Carcinoma. *Current Oncology Reports*.

[4] Thawani R., Kim M. S., Arastu A. (2023). The Contemporary Management of Cancers of the Sinonasal Tract in Adults. *CA: A Cancer Journal for Clinicians*.

[5] Chang M. H., Kuo Y. J., Ho C. Y., Kuan E. C., Lan M. Y. (2019). Metastatic Tumors of the Sinonasal Cavity: A 15-Year Review of 17 Cases. *Journal of Clinical Medicine*.

[6] Mendenhall W. M., Mendenhall C. M., Riggs C. E., Villaret D. B., Mendenhall N. P. (2006). Sinonasal Undifferentiated Carcinoma. *American Journal of Clinical Oncology*.

[7] López F., Devaney K. O., Hanna E. Y., Rinaldo A., Ferlito A. (2016). Metastases to Nasal Cavity and Paranasal Sinuses. *Head & Neck*.

[8] Abi-Fadel F., Ayaz A., Sundaram K., Smith P. (2012). Paranasal Sinus Involvement in Metastatic Carcinoma. *Journal of Neurological Surgery Reports*.

[9] Ali E. H., Mengesha M. W. (2024). Sinonasal Adenocarcinoma Presented as a Giant Anterior Cranial Fossa Mass: A Case Report and Review of the Literature. *Journal of Medical Case Reports*.

[10] Kumar S., Ranjan S., Mittal A., Mammen K. J., Navariya S. C., Bhirud D. P. (2020). Epistaxis Presenting as Sentinel Feature of Metastatic Renal Cell Carcinoma: A Case Report and Review of Literature. *Journal of Family Medicine and Primary Care*.

[11] S Serra A., Caltabiano R., Giorlandino A. (2017). Nasal Metastasis as the First Manifestation of a Metachronous Bilateral Renal Cell Carcinoma. *Pathologica*.

[12] Nayak D. R., Pujary K., Ramnani S., Shetty C., Parul P. (2006). Metastatic Renal Cell Carcinoma Presenting With Epistaxis. *Indian Journal of Otolaryngology and Head & Neck Surgery*.

[13] Hess A. O., Terry R. S., Lobo B. C., Justice J. M. (2023). Sinonasal and Skull Base Metastatic Renal Cell Carcinoma: A Case Series. *Cureus*.

[14] Sikdar A., Khan S., Agarwal R., Phatak S., Bhagat P., Patidar R. (2023). Metastatic Renal Cell Carcinoma: An Enigmatic Nasal Mass. *Indian Journal of Otolaryngology and Head & Neck Surgery*.

[15] Nason R., Carrau R. L. (2004). Metastatic Renal Cell Carcinoma to the Nasal Cavity. *American Journal of Otolaryngology*.

[16] Ziari M., Shen S., Amato R. J., Teh B. S. (2006). Metastatic Renal Cell Carcinoma to the Nose and Ethmoid Sinus. *Urology Times*.

[17] Kumar R., Sikka K., Kumar R., Chatterjee P. (2014). Nephrogenic Epistaxis. *Singapore Medical Journal*.

[18] Doğan S., Can İ. H., Sayn M. (2009). The Nasal Septum: An Unusual Presentation of Metastatic Renal Cell Carcinoma. *Journal of Craniofacial Surgery*.

[19] Parida P. K. (2012). Renal Cell Carcinoma Metastatic to the Sinonasal Region: Three Case Reports With a Review of the Literature. *Ear, Nose & Throat Journal*.

[20] Hefer T., Joachims H. Z., Golz A. (1994). Metastatic Renal Cell Carcinoma to the Nose. *European Archives of Oto-Rhino-Laryngology*.

[21] Goger Y. E., Piskin M. M., Balasar M., Kilinc M. (2013). Unusual Presentation of Renal Cell Carcinoma: Gluteal Metastasis. *Case Reports in Urology*.

[22] Mueller T. J., Wu H., Greenberg R. E. (2004). Cutaneous Metastases From Genitourinary Malignancies. *Urology Times*.

[23] Gulati A., Kaushal V., Kaushik R., Mahajan P., Jaswal K. S. (2015). Cutaneous Metastasis of Renal Cell Carcinoma Masquerading as Scrotal Growth. *Indian Journal of Surgery*.

[24] Huber J., Ihrig A., Winkler E. (2015). Interdisciplinary Counseling Service for Renal Malignancies: A Patient-Centered Approach to Raise Guideline Adherence. *Urologic Oncology: Seminars and Original Investigations*.

[25] Wheless S. A., McKinney K. A., Zanation A. M. (2010). A Prospective Study of the Clinical Impact of a Multidisciplinary Head and Neck Tumor Board. *Otolaryngology–Head and Neck Surgery*.

